# Three-dimensional organoid culture reveals involvement of Wnt/β-catenin pathway in proliferation of bladder cancer cells

**DOI:** 10.18632/oncotarget.24308

**Published:** 2018-01-24

**Authors:** Takahiro Yoshida, Nikolai A. Sopko, Max Kates, Xiaopu Liu, Gregory Joice, David J. McConkey, Trinity J. Bivalacqua

**Affiliations:** ^1^ The James Buchanan Brady Urological Institute and Department of Urology, Johns Hopkins School of Medicine, Baltimore, Maryland, USA; ^2^ The Johns Hopkins Greenberg Bladder Cancer Institute, Baltimore, Maryland, USA

**Keywords:** Wnt, β-catenin, bladder cancer, organoid, spheroid

## Abstract

There has been increasing awareness of the importance of three-dimensional culture of cancer cells. Tumor cells growing as multicellular spheroids in three-dimensional culture, alternatively called organoids, are widely believed to more closely mimic solid tumors *in situ*. Previous studies concluded that the Wnt/β-catenin pathway is required for regeneration of the normal urothelium after injury and that β-catenin is upregulated in human bladder cancers, but no clear evidence has been advanced to support the idea that the Wnt/β-catenin pathway is directly involved in deregulated proliferation and the other malignant characteristics of bladder cancer cells. Here we report that the Wnt/β-catenin pathway activator, CHIR99021, promoted proliferation of established human bladder cancer cell lines when they were grown in organoid culture but not when they were grown in conventional adherent cultures. CHIR99021 activated Wnt/β-catenin pathway in bladder cancer cell lines in organoid culture. CHIR99021 also stimulated proliferation and the Wnt/b-catenin pathway in primary human bladder cancer organoids. RNAi-mediated knockdown of β-catenin blocked growth of organoids. The effects of CHIR99021 were associated with decreased expression of the urothelial terminal differentiation marker, cytokeratin 20. Our data suggest that the Wnt/β-catenin pathway is required for the proliferation of bladder cancer cells in three-dimensional organoid culture and provide a concrete example of why organoid culture is important for cancer research.

## INTRODUCTION

Bladder cancer is the 9th most common cancer worldwide [1]. In the United States, 79000 new cases and 17000 cancer deaths were estimated to have occurred in 2017 [2]. Approximately 75% of the patients are diagnosed with non-muscle invasive bladder cancer, which is not immediately life-threatening but frequently recurs, whereas muscle invasive bladder cancer progresses rapidly and poses a significant risk of mortality [3–5]. Recent advances in the molecular characterization of bladder cancer deciphered intrinsic subtypes associated with prognosis as well as response to systemic therapy [6–10], providing insight into the molecular heterogeneity of the disease and candidate therapeutic targets for clinical intervention [6–10].

Targetable mechanisms required for cancer growth can also be revealed by examining the similarities between cancer and its normal benign tissue of origin [11, 12]. Signaling pathways, cellular responses to the microenvironment, differentiation process, and cellular migration are commonly shared by both cancers and normal tissues. Putative cancer stem cells and normal tissue stem cells are also phenotypically similar and are both endowed with self-renewal potential and the capacity for differentiation, creating hierarchically organized tissues [13, 14]. Among such shared properties, the Wnt pathway, which governs stem cell behavior in normal tissue, has also been implicated in pathogenesis of malignant tumors [11, 15]. Of particular importance is the canonical Wnt pathway, which acts in large part via the transcriptional co-activator, β-catenin [15]. The canonical Wnt/β-catenin pathway is active when β-catenin is hypophosphorylated, which allows β-catenin to escape proteosomal degradation, accumulate, and translocate to the nucleus, where it interacts with co-transcriptional factors to activate Wnt target genes [16].

With respect to the normal bladder, the Wnt/β-catenin pathway was reported to be required for regeneration of the normal urothelium after injury in mice [17, 18]. Thus, it seems likely that the Wnt/β-catenin pathway should also be involved in pathogenesis of bladder cancer. Indeed, β-catenin was shown to be upregulated in human bladder cancer specimens as compared to normal urothelium [19–21]. Transgenic mice, which overexpressed β-catenin under the promoter of uroplakin II developed benign hyperplasia in urothelium [19]. Although preceding studies have indirectly suggested the involvement of Wnt/β-catenin pathway in malignant behavior of bladder cancer cells [21–23], whether or not the pathway plays a central role in proliferation in bladder cancer cells is still unclear.

There has been increasing awareness of the importance of three-dimensional culture for capturing critical biological properties in cancer cells maintained *ex vivo*. More specifically, multicellular spheroids, or “organoids”, are thought to more closely mimic solid tumors with regard to cell-cell interactions, hypoxia, drug penetration, and nutrition gradients [24, 25]. Organoids generated from cancer cell lines alter their molecular phenotypes and sensitivity to drugs compared to their parental cells grown in conventional adherent culture [26–28]. Our group also found that organoids generated from bladder cancer cell lines had increased expression of ∆Np63α, a transcription factor that appears to be important for regulating “stemness” in a variety of epithelial tissues [29]. Thus, we hypothesized that organoids derived from bladder cancer cells might also exhibit differences in the expression of other stem cell pathways as compared to the same cells maintained in adherent culture.

In this study we compared the effects of the Wnt/β-catenin pathway activator, CHIR99021 (CHIR), on proliferation in established bladder cancer cell lines maintained in organoid culture or standard two-dimensional adherent cultures, and we also characterized the effects of CHIR on proliferation and pathway activation in primary patient-derived organoids. The results reveal that three-dimensional culture is required for Wnt/β-catenin pathway-mediated proliferation in bladder cancer cells.

## RESULTS

### CHIR suppresses proliferation of bladder cancer cell lines in conventional adherent culture, but increases proliferation in three-dimensional organoid culture

**Figure 1:**
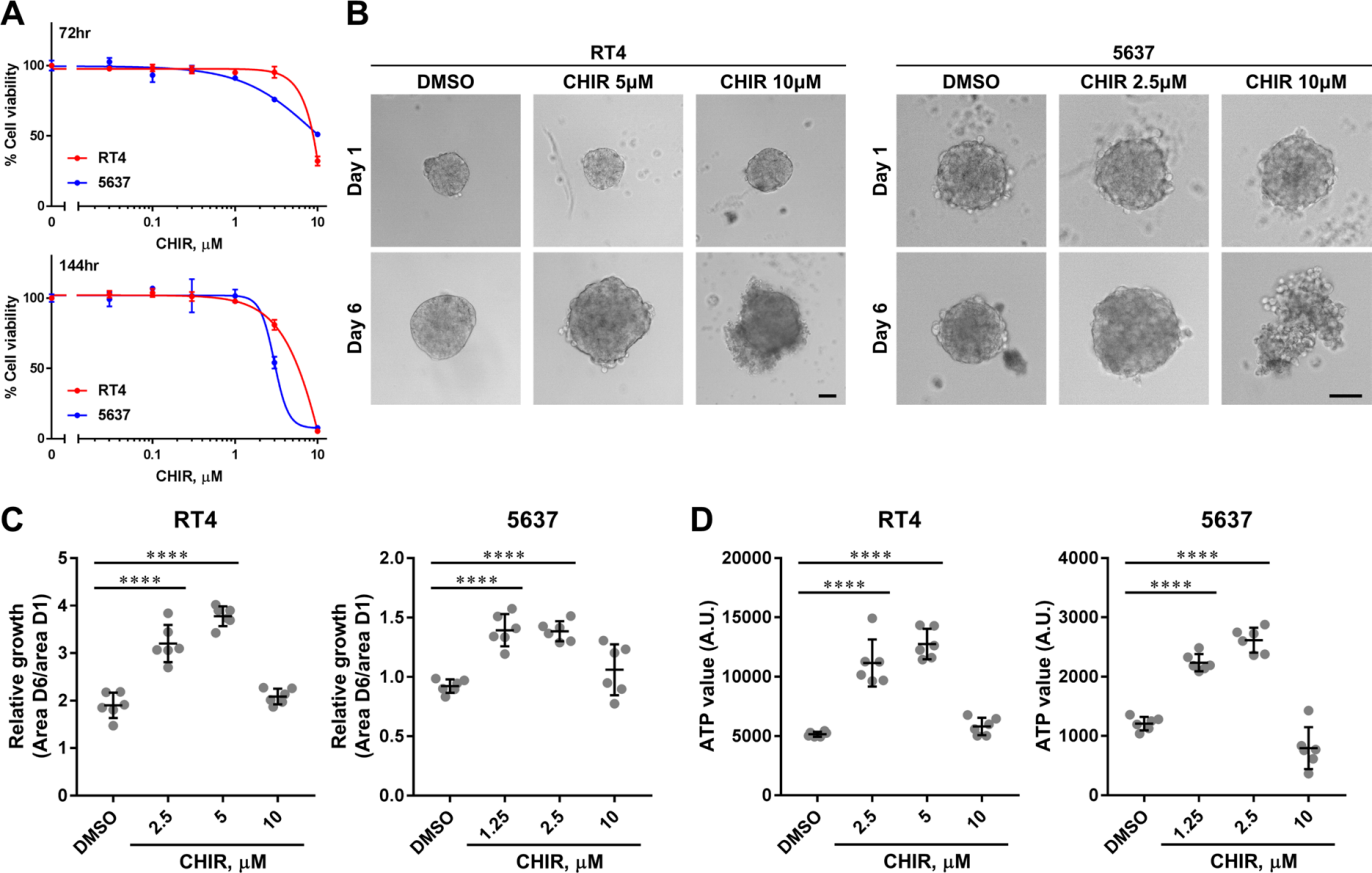
CHIR inhibits proliferation of bladder cancer cell lines in adherent culture but promotes proliferation in organoid culture (**A**) Dose-response curves of CHIR on proliferation of RT4 and 5637 for the indicated duration. (**B**) Representative images of RT4- and 5637-derived organoids at day 1 and day 6 treated with DMSO or CHIR of the indicated concentration. Scale bar, 100 µm. (**C**) Relative growth of organoids was calculated by dividing area at day 6 by that at day 1, and depicted in the plot. (**D**) Viability of organoids was measured by quantifying the amount of ATP, and depicted in the plot. Each dot represents relative growth or viability of one organoid. ^****^P ≤ 0.0001.

In order to assess the effects of Wnt/β-catenin pathway activation on proliferation, we used the small molecule CHIR, a Wnt/β-catenin pathway activator [30, 31]. CHIR can activate Wnt/β-catenin pathway by inhibiting GSK3 and subsequent phosphorylation and degradation of β-catenin [30]. RT4 and 5637 were selected to test as representative luminal and basal bladder cancer cell lines based on recent identification of molecular subtypes of human bladder cancer, respectively [7, 32]. When RT4 and 5637 were treated with DMSO or CHIR in conventional adherent culture, CHIR exhibited suppressive effects on proliferation in a dose-dependent manner (Figure 1A). In a previous study, we found that bladder cancer cells changed their molecular phenotypes when they were cultured as organoids in suspension [29]. We hypothesized that they could also change their functional phenotypes, and tested the effect of CHIR on organoid growth patterns. When RT4 was cultured as organoids, they grew over time and this growth was enhanced by CHIR treatment, although the highest dose was not beneficial to the growth of the organoids (Figure 1B, 1C). Interestingly, 5637-derived organoids could not grow over time without stimulation even though 5637 can proliferate more rapidly than RT4 in adherent culture (Figure 1B, 1C). However, as was seen in RT4-derived organoids, the growth of 5637-derived organoids was induced by CHIR treatment and the highest dose caused disorganization of the organoids (Figure 1B, 1C). Incorporation of propidium iodide in cells drastically increased in organoids treated with 10 µM of CHIR, while only a few cells incorporated propidium iodide with lower concentration of CHIR, indicating CHIR at high dose induced cell death (Supplementary Figure 1). When the viability of cells was measured by the amount of ATP, the values increased with the concentration of CHIR except for the highest dose, corresponding to the results of the growth assay (Figure 1D). The ATP value correlated with the area of organoids (Supplementary Figure 2). These indicated that the growth of organoids was paralleled with proliferation of bladder cancer cells. Taken together, the data show that CHIR promoted the proliferation of both bladder cancer cell lines within a certain range of concentrations in organoid cultures but not in conventional adherent cultures.

### CHIR activates the Wnt/β-catenin pathway in bladder cancer lines in organoid culture

**Figure 2:**
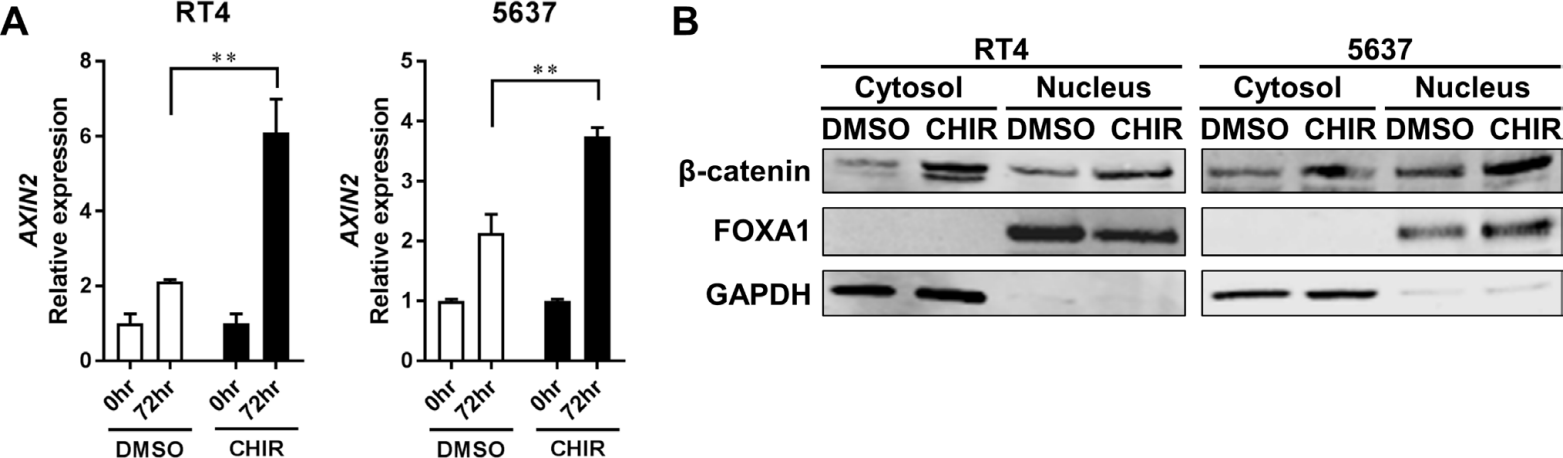
CHIR activates Wnt/β-catenin pathway in bladder cancer cell line-derived organoids (**A**) Bar graphs show the relative gene expression of AXIN2 by qRT-PCR in RT4- and 5637-derived organoids treated for 72 hours with DMSO or CHIR of 5 and 2.5 µM for RT4 and 5637, respectively. GAPDH was used as a reference gene, and relative expression was calculated by the 2 ΔΔCT method. ^**^P ≤ 0.01. (**B**) Western blot analysis shows protein levels of β-catenin, FOXA1, and GAPDH in RT4- and 5637-derived organoids. Organoids were treated for 24 hours with DMSO or CHIR of 5 and 2.5 µM for RT4 and 5637, respectively. Cytoplasmic and nuclear proteins were separated and analyzed. GAPDH and FOXA1 were used as a loading control for cytoplasmic and nuclear protein, respectively.

Next, we investigated whether the Wnt/β-catenin pathway was activated following CHIR treatment in bladder cancer cell lines in organoid culture. AXIN2, which is a gene that is induced following activation of the Wnt/β-catenin pathway [30, 31], was significantly upregulated by CHIR treatment in RT4- and 5637-derived organoids compared to DMSO-treated control (Figure 2A, Supplementary Figure 3). Notably, AXIN2 was also upregulated over time even without CHIR treatment in both RT4- and 5637-derived organoids (Figure 2A). To confirm activation of Wnt/β-catenin pathway at a protein level, western blotting analysis for β-catenin was conducted [30, 31]. Immnoblots confirmed that CHIR treatment increased the amount of β-catenin in both cytoplasm and nucleus of RT4- and 5637-derived organoids (Figure 2B). CHIR treatment also upregulated AXIN2 expression and increased the amount of β-catenin in RT4 and 5637 in adherent culture (Supplementary Figure 4). Collectively, these data indicated that the Wnt/β-catenin pathway was activated by CHIR treatment in bladder cancer cell lines in both adherent and organoid cultures.

### CHIR99021 enhances proliferation of primary human bladder cancer cells with activation of Wnt/β-catenin pathway in organoid culture

**Figure 3:**
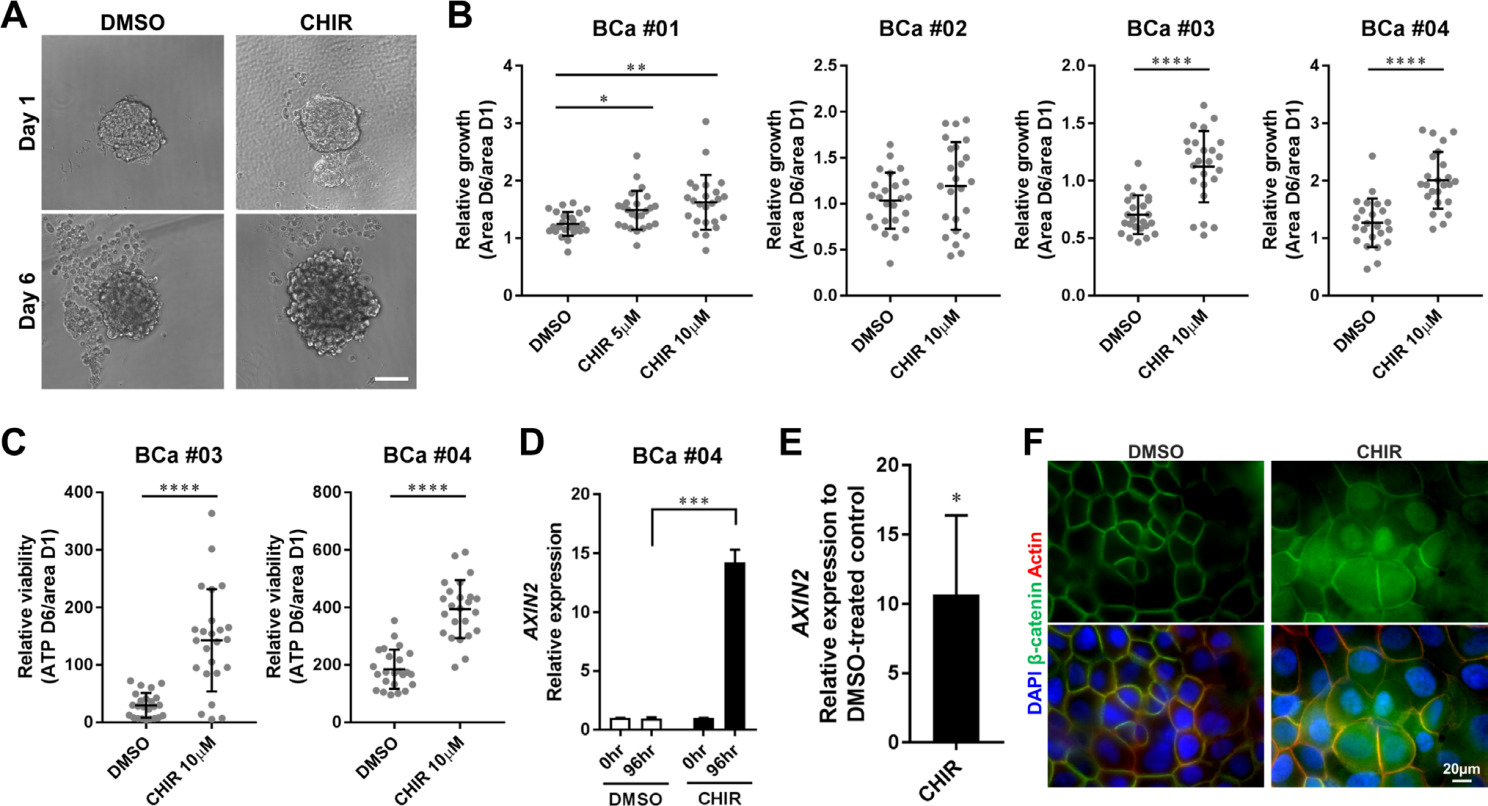
CHIR enhances proliferation of primary bladder cancer cells in organoid culture with activation of Wnt/β-catenin pathway (**A**) Representative images of primary cell organoids (BCa #01) at day 1 and day 6 treated with DMSO or CHIR of 10 µM. Scale bar, 100 µm. (**B**) Relative growth of organoids from 4 patient samples was calculated by dividing area at day 6 by that at day 1, and depicted in the plot. (**C**) Relative viability of organoids from 2 patient samples was measured by dividing the amount of ATP at day 6 by the area of organoid at day 1, and depicted in the plot. Each dot represents relative growth or viability of one organoid. (**D**) Bar graphs show the relative gene expression of AXIN2 by qRT-PCR in primary cell organoids (BCa #04) treated with DMSO or CHIR of 10 µM for 96 hours. GAPDH was used as a reference gene, and relative expression was calculated by the 2 ΔΔCT method. (**E**) A bar graph shows the relative gene expression of AXIN2 by qRT-PCR in primary cell organoids of 3 pooled patient samples (BCa #02, #03, #04). The result was normalized to the expression of DMSO-treated organoids.^*^P ≤ 0.05. ^**^P ≤ 0.01. ^***^P ≤ 0.001. ^****^P ≤ 0.0001. (**F**) The expression and subcellular localization of β-catenin in primary cell organoids (BCa #04) treated with DMSO or 10 µM CHIR for 72 hours was detected by immunofluorescence staining. Nuclei and actin fibers were counterstained with DAPI and ActinRed, respectively. Scale bar 20 µm.

Immortalized cancer cell lines are highly selected cell populations that have been cultured for years *ex vivo,* and they consequently lose some of the original characteristics of the tumors they were derived from [33]. Thus, we prepared organoids from surgically resected primary human bladder tumors using the CTOS method to evaluate the effects of the Wnt/β-catenin activator in minimally selected cancer cells. The CTOS method enables preparation of organoids, which consist of highly purified cancer cells and retain characteristics of the original tumor [34–36]. When primary organoids were treated with CHIR, their growth rates were significantly greater than DMSO-treated controls in 3 out of 4 samples examined (Figure 3A, 3B). Even in the one (BCa #02), which did not show a significant difference, several organoids could grow more in response to CHIR than control (Figure 3B). We observed more variability among primary organoids than RT4- and 5637-derived organoids in terms of growth (Figure 1C, 3B), indicating the presence of heterogeneity within primary tissue. The amount of ATP was also significantly elevated in CHIR-treated organoids compared to DMSO-treated control (Figure 3C), and the ATP value correlated with the area of organoids (Supplementary Figure 5), suggesting that the growth of primary organoids was concurrent with proliferation of cancer cells. In aggregate, CHIR also enhanced proliferation of primary bladder cancer cells in organoid culture.

We also investigated status of Wnt/β-catenin pathway in primary organoids treated with CHIR. AXIN2 expression did not change over time with DMSO treatment but was significantly upregulated with CHIR treatment in primary organoids prepared from BCa #04 (Figure 3D). Analysis of 3 pooled patient samples confirmed upregulation of AXIN2 expression with CHIR treatment in primary organoids (Figure 3E). Whole mount staining of organoids demonstrated increased expression of β-catenin in cytoplasm and translocation of β-catenin into nuclei in primary organoids treated with CHIR (Figure 3F). Collectively, the data confirm that CHIR activated the Wnt/β-catenin pathway in primary bladder cancer cells in organoid culture.

### β-catenin is required for growth of bladder cancer organoids

**Figure 4:**
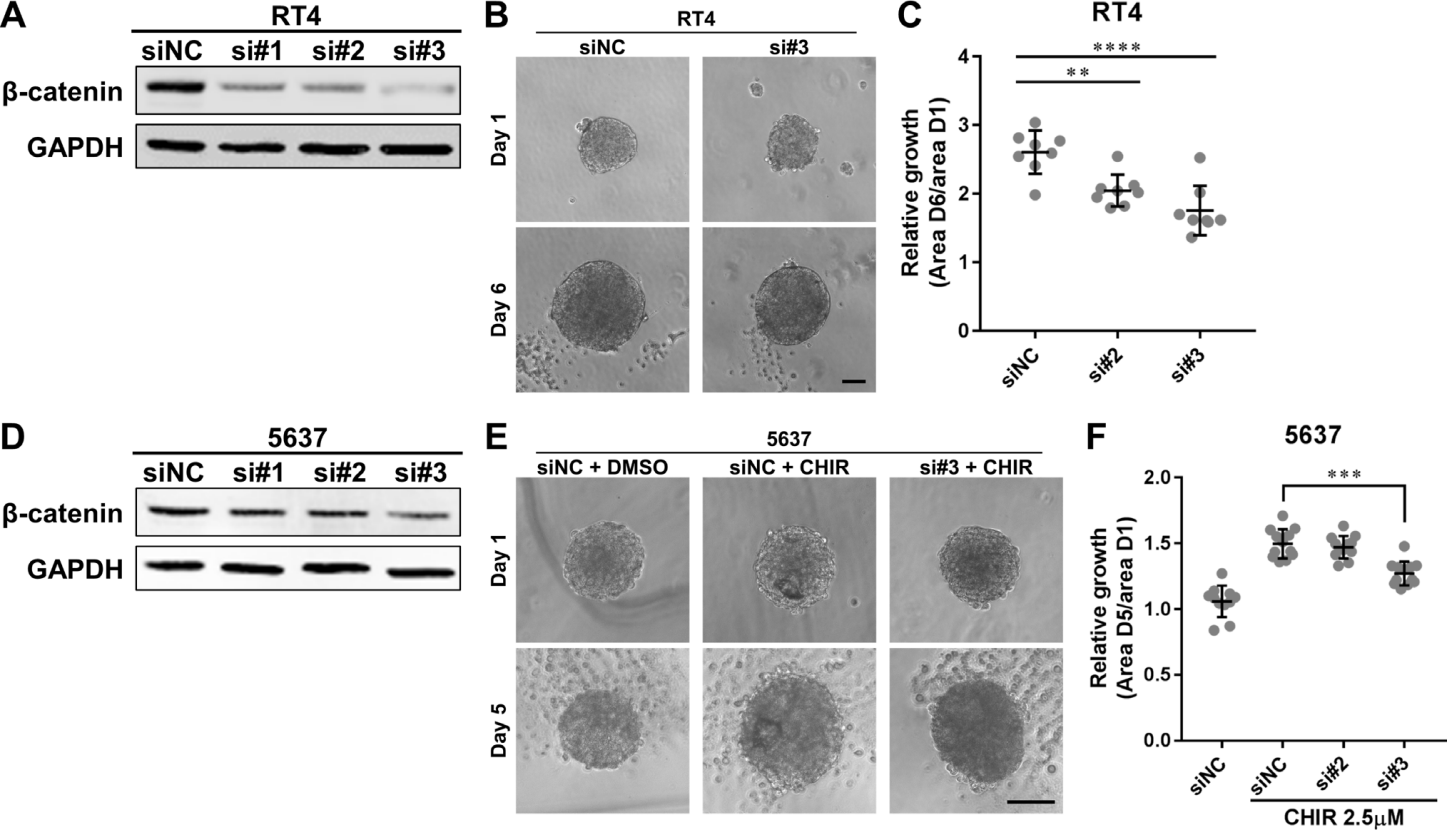
β-catenin is required for growth of bladder cancer organoids (**A**, **D**) Western blot of β-catenin in RT4 and 5637 transiently transfected with siRNAs against β-catenin. Protein was extracted 48 hours after transfection. GAPDH was used as a loading control. NC, negative control. (**B**) RT4 was transiently transfected with siRNAs and used to generate organoids. Images show representative RT4-derived organoids after transfection at day 1 and 6. Scale bar, 100 µm. (**C**) Relative growth of RT4-derived organoids after transfection was quantified and plotted. (**E**) 5637 was transiently transfected with siRNAs and used to generate organoids. The organoids were subsequently treated with 2.5 µM of CHIR. Images show representative 5637-derived organoids after transfection and treatment at day 1 and 5. Scale bar, 100 µm. (**F**) Relative growth of 5637-derived organoids after transfection and treatment was quantified and plotted. Each dot represents relative growth of one organoid. ^**^P ≤ 0.01. ^***^P ≤ 0.001. ^****^P ≤ 0.0001.

Since a small molecule like CHIR can have off-target effects, we adopted gene silencing approach against β-catenin to clarify more specifically whether Wnt/β-catenin pathway is involved in proliferation of bladder cancer cells. Western blotting confirmed efficient knockdown of β-catenin in RT4 and 5637 after transfection of the siRNAs (Figure 4A, 4D). Knockdown of β-catenin had a slight effect on proliferation of RT4 and 5637 in adherent culture (Supplementary Figure 6). Since 5637-derived organoids did not grow without CHIR, we evaluated the effect of β-catenin on intrinsic growth in RT4-derived organoids. Growth of β-catenin-knockdowned RT4-derived organoids was significantly less than that of control organoids (Figure 4B, 4C). We also examined whether β-catenin was required in CHIR-induced growth of organoids. Growth of β-catenin-silenced 5637-derived organoids was significantly smaller than that of control organoids with CHIR treatment (Figure 4E, 4F). Similarly, pharmaceutical inhibition of the β-catenin-mediated transcription using ICG-001 suppressed intrinsic growth of RT4-derived organoids and CHIR-induced growth of 5637-derived organoids (Supplementary Figure 7). The ATP value correlated with the area of organoids (Supplementary Figure 8). On the other hand, when β-catenin was overexpressed in 5637, the growth of the organoids did not change (Supplementary Figure 9). Taken together, β-catenin was required but not sufficient for growth of bladder cancer organoids.

### CK20 is less expressed over CHIR-induced proliferation in bladder cancer organoids

**Figure 5:**
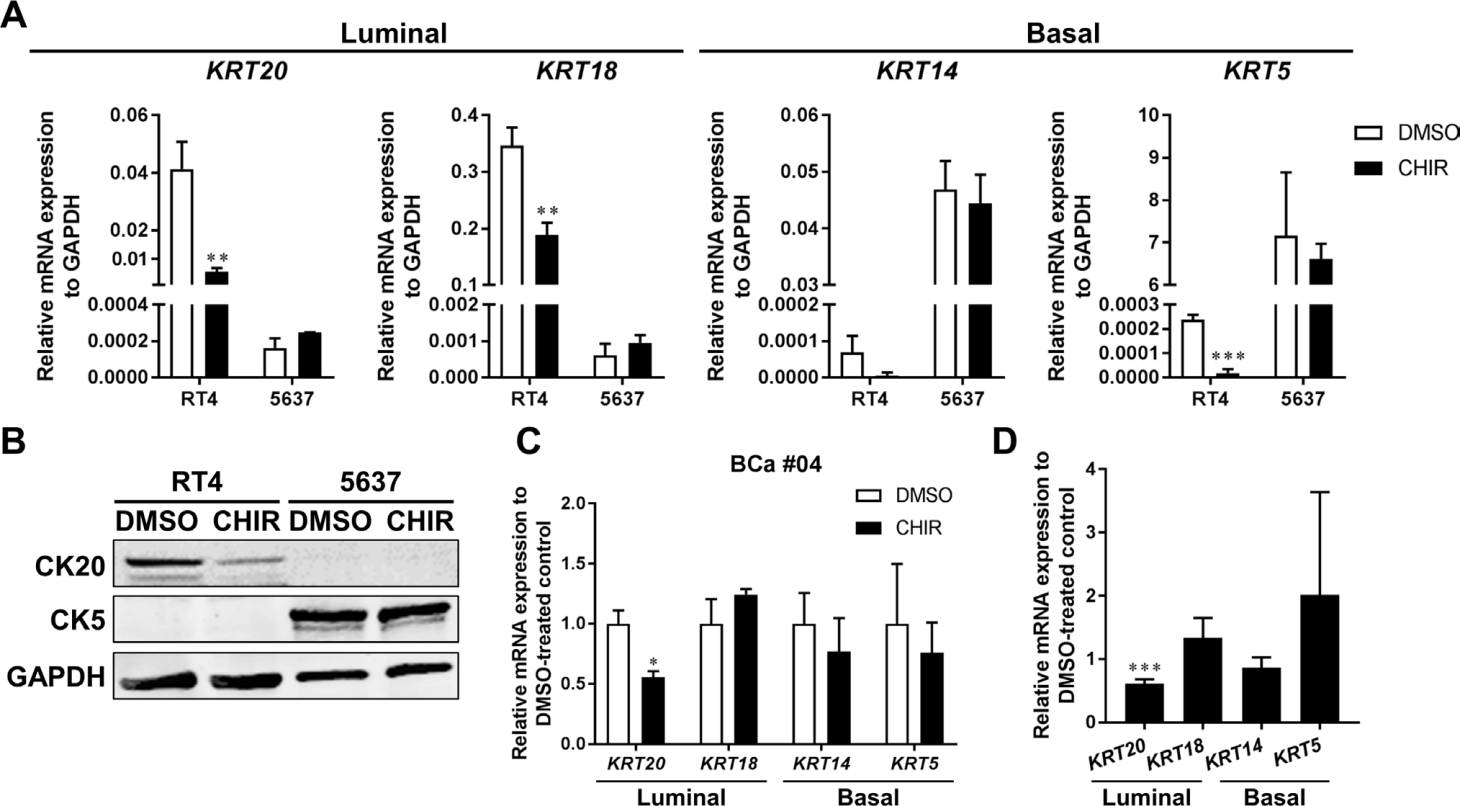
CK20 is less expressed in CHIR-stimulated bladder cancer organoids (**A**) qRT-PCR shows mRNA expression of luminal markers (CK20, CK18) and basal markers (CK14, CK5) in RT4- and 5637-derived organoids treated with DMSO or CHIR for 72 hours. GAPDH was used as a reference gene. (**B**) Western blot analysis demonstrates protein levels of CK20 and CK5 in RT4- and 5637-derived organoids. Organoids were treated for 72 hours with DMSO or CHIR of 5 and 2.5 µM for RT4 and 5637, respectively. GAPDH was used as a loading control. (**C**) Bar graphs show the relative gene expression of CK20, CK18, CK14, and CK5 by qRT-PCR in primary cell organoids (BCa #04) treated with DMSO or CHIR of 10 µM for 96 hours. GAPDH was used as a reference gene, and relative expression was calculated by the 2 ΔΔCT method. (**D**) Bar graphs show the relative gene expression of CK20, CK18, CK14, and CK5 by qRT-PCR in primary cell organoids of 3 pooled patient samples (BCa #02, #03, #04). The results were normalized to the expression of DMSO-treated organoids. ^*^P ≤ 0.05. ^**^P ≤ 0.01. ^***^P ≤ 0.001.

Urothelium consists of multilayered urothelial cells which encompasses spectrum of differentiation status [37]. Among them, basal cells are capable of regenerating all cell types within the urothelium in response to Wnt/β-catenin signaling in a bladder injury model in mice [17]. Basal cells also could give rise to muscle invasive cancer accompanied by transition of their differentiation status in carcinogen-induced bladder cancer in mice [18, 38]. Stimulatory factors possibly derived from stromal cells which could enhance urothelial differentiation restrained bladder cancer progression [39]. Therefore, we examined the differentiation status of bladder cancer organoids over CHIR-induced growth. qPCR revealed that luminal markers, CK20 and CK18, were expressed more in RT4 than 5637, while basal markers, CK14 and CK5, were more in 5637 than RT4 (Figure 5A). The result was in line with the finding that RT4 and 5637 were luminal and basal phenotypes, respectively [32]. CK20, CK18, and CK14 were significantly less expressed in CHIR-treated RT4-derived organoids than DMSO-treated controls, while all 4 genes examined remained unchanged in 5637-derived organoids (Figure 5A). Western blotting analysis also showed that CK20 was detectable in RT4 but not in 5637, and CK5 was in 5637 but not in RT4 (Figure 5B). CK20 was expressed at lower levels at the protein level in CHIR-treated RT4-derived organoids as compared to DMSO-treated controls (Figure 5B). qPCR revealed that CK20 was expressed at lower levels in CHIR-treated primary organoids as well (Figure 5C, 5D). Collectively, lower expression of CK20 was consistently observed in CHIR-treated bladder cancer organoids than in DMSO-treated controls.

## DISCUSSION

In the present study, we showed that bladder cancer cell lines exhibited different responses to the Wnt/β-catenin pathway activator CHIR depending on the culture condition. CHIR had a negative effect on proliferation of bladder cancer cell lines in adherent culture but increased proliferation in organoid culture, although activation of the Wnt/β-catenin pathway was observed in both culture conditions. CHIR could also enhance proliferation of primary human bladder cancer cells in organoid culture with activation of Wnt/β-catenin pathway. Furthermore, β-catenin was shown to be required for growth of bladder cancer organoids. Lastly, CK20, which is a marker for terminal differentiation, was downregulated in CHIR-treated organoids as compared to DMSO controls.

Experimental findings must be interpreted with caution when the effect of an inhibitor is evaluated on proliferation of cancer cells *in vitro*, because, as we demonstrated, cancer cells can completely change their responses to a drug when they are cultured as organoids. For example, CHIR was reported to attenuate proliferation and induce apoptosis of cancer cells in adherent culture as a possible therapeutic agent [40–42]. However, given the fact that we observed diverse findings of bladder cancer cell lines to CHIR between adherent and organoid culture as it related to proliferation, all experimental results evaluating the effect of CHIR on cell viability should be studied in both culture conditions.

Further characterization of what makes organoids more responsive to Wnt/β-catenin pathway in bladder cancer cell lines is warranted. We previously reported that bladder cancer cells in organoid culture increased the amount of ∆Np63α protein [29], which positively controls basal cell gene signature in bladder cancer cells [7]. Basal cells were the main targets for upregulation of the Wnt/β-catenin pathway in a bladder injury model in mice [17]. Thus, we hypothesized that basal cell program would be induced in organoid culture compared to adherent culture. However, when we examined mRNA expression of CK14 and CK5 by qPCR, they were more upregulated in 5637 in organoid culture than in adherent culture, but not in RT4 (data not shown). Further investigation is required to clarify the mechanism underlying different response to CHIR in organoid culture.

We observed that CK20, which is a marker for terminal differentiation and expressed in the inner most layer of urothelium [37], was expressed at lower levels in CHIR-stimulated organoids of bladder cancer as compared with controls. Bladder cancer consists of heterogeneous populations that include a small subset of cells, which are more tumorigenic and marked by stem and basal cell markers, and the rest of the cells, which are less tumorigenic and express higher levels of differentiation markers [38, 43, 44]. Indeed, CK20-expressing cells were heterogeneously observed in RT4-derived organoids (data not shown). Interestingly, Wnt/β-catenin signaling was enriched more in basal-like cancer cells within a tumor [43]. In addition, cells expressing basal markers are progenitor cells in *ex vivo* urothelial cell culture [17, 18]. Considering those findings, CHIR would stimulate proliferation of basal-like cancer cells within an organoid, which would lead to less expression of CK20. Alternatively, there is still possibility that CHIR could transform luminal-like cancer cells into basal-like ones expressing less CK20. Basal markers examined remained unchanged in CHIR-treated organoids of bladder cancer. At a protein level, the amount of CK5 did not change in 5637-derived organoids with the treatment of CHIR. Since immunohistochemistry showed that CK5 expressed homogeneously in cells of 5637-derived organoids (data not shown), it is still unclear whether only basal-like cells can proliferate in response to CHIR. Additional studies are necessary to delineate heterogeneity of bladder cancer cells with regard to response to the Wnt/β-catenin pathway.

In the present study, we have shown that β-catenin was necessary but not sufficient for growth of bladder cancer organoids, although CHIR could enhance growth of bladder cancer organoids with increased expression of β-catenin. CHIR is a GSK3 inhibitor and GSK3 can interact with not only Wnt/β-catenin pathway but also other pathways like Notch signaling pathway [45]. For example, CHIR upregulates both β-catenin and Notch3 proteins in non-small lung cancer cell lines [46], although functional relevance of Notch3 in bladder cancer is unknown compared to Notch1 and Notch2 [47]. Other signaling pathways than Wnt/β-catenin pathway will be also involved in CHIR-induced growth of bladder cancer organoids.

Wnt/β-catenin pathway was reported to be required for the proliferation of normal urothelial cells during repair after injury in mice [17, 18]. The pathway is also indispensable in urothelial explant tissue growth [18]. Considering that a signaling pathway in normal tissue can be commonly observed in cancer cells, our results suggest that CHIR also might be able to enhance proliferation of normal urothelial cells.

One of the limitations of the present study is that the cancer cells used in this study were mostly derived from early stage of bladder cancer. RT4 and 5637 retain epithelial characteristics and are considered less aggressive than mesenchymal phenotypes. Besides, the method used to prepare primary organoids is difficult to apply for muscle-invasive cancer. Thus, additional studies are needed to evaluate the role of Wnt/β-catenin pathway in proliferation of more aggressive bladder cancer cells. Furthermore, the mechanisms underlying different responses to CHIR between organoid and adherent culture are still elusive. We are currently designing a series of experiments to perform transcriptome analysis for more comprehensive understanding of their phenotypic change between culture conditions. It is also intriguing to examine involvement of Wnt/β-catenin signaling in bladder cancer cells in *in vivo* experiments. We hypothesize that Wnt ligands could come from microenvironment surrounding cancer cells in a paracrine manner in bladder tumor, because sequencing studies in patient specimens was not supportive for occurrence of activating somatic alterations in the pathway [10], and Wnt ligands could be released from a stromal compartment in a bladder injury model in mice [17]. We are planning to perform *in vivo* experiments using an appropriate model based on our hypothesis to further understand a role of Wnt/β-catenin pathway in bladder tumor.

In conclusion, we showed for the first time that Wnt/β-catenin pathway was directly involved in proliferation of bladder cancer cells by using organoid culture, which could not be found in conventional adherent culture. Different responses of cancer cells to CHIR between organoid and adherent culture underscores importance of organoid culture for drug screening *in vitro*. The Wnt/β-catenin pathway may be a potential target for the treatment of a subset of bladder cancer.

## MATERIALS AND METHODS

### Ethics statement

The institutional review board at the Johns Hopkins School of Medicine approved the protocol for the research use of human bladder cancer specimens surgically resected from patients. Patients gave written informed consent for their use. All samples used in the study were collected from patients treated at the Department of Urology, the Johns Hopkins Hospital.

### Cells and cell culture

Bladder cancer cell lines, RT4 and 5637 were purchased from the American Type Culture Collection (ATCC, Manassas, VA, USA) in November 2015. Cell lines were tested and authenticated by short tandem repeat analysis by the ATCC. Cells were cultured at 37° C under 5% CO_2_ in RPMI 1640. Organoids from cell lines were prepared by an aggregation-based method [29, 48]. Briefly, 2 × 10^5^ or 1000 cells (respectively) were seeded in 6- or 96-well plates coated with poly-2-hydroxyethyl methacrylate (Sigma). Organoids were formed 24 hours after seeding and used in further experiments. Organoids of primary bladder cancer cells were prepared according to the cancer tissue-originated spheroid (CTOS) method [34–36]. Briefly, surgically resected samples were mechanically dissociated and partially digested with liberase DH (Roche Applied Science, Mannheim, Germany). Clusters of cells were collected and cultured in DMEM/F-12 GlutaMAX with bovine serum albumin and 2-mercaptoethanol, which were supplied with StemPro hESC human embryonic stem cell culture medium (Invitrogen, Carlsbad, CA, USA). All bladder tumor samples were confirmed as urothelial carcinoma by institutional pathologists. CHIR was purchased from STEMCELL technologies (Vancouver, Canada), reconstituted in dimethyl sulfoxide (DMSO), and stored at –20° C. CHIR added to culture medium at the indicated concentration.

### Viability assay and growth assay

For viability assay in adherent culture, 3000 and 1500 cells of RT4 and 5637 were seeded in a 96-well tissue-treated plate in triplicate, respectively. Twenty-four hours after seeding, cells were treated with DMSO or CHIR for 72 or 144 hours. Cell viability was then measured using CellTiter-Glo Luminescent Cell Viability Assay (Promega) and FLUOstar OPTIMA (BMG Labtech) according to manufactures’ protocols. Following this, dose response curves of CHIR were depicted using GraphPad Prism 7 (GraphPad Sofrware, San Diego, CA, US) with non-linear (curve fit) regression algorithms.

To measure growth and viability in organoid cultures, organoids were incubated with DMSO or CHIR in 96-well plates for 6 days. Images of organoids were collected under a microscope at days 1 and 6, and the areas occupied by the organoids were measured using Image J software (National Institutes of Health, Bethesda, MD). Relative growth rates were calculated by dividing the area of organoids at day 6 by that at day 1. For viability assays, ATP levels at day 6 were measured as described above. For viability assay of primary organoids, ATP value at day 6 was corrected for the organoid area at day 1, since it was difficult to precisely collect the same size of primary cell organoids at day 1 and the area of organoids correlates with the ATP value [49].

### Western blotting

Organoids were lysed with RIPA buffer (Sigma-Aldrich, St. Louis, MO, USA) with EDTA-free protease inhibitor cocktail (Roche Applied Science, Mannheim, Germany). Nuclear and cytosolic protein was fractionated using Nuclear/Cytosol Fractionation Kit (BioVison). Subsequently, protein concentration was measured by BCA Protein Assay Kit (Thermo Fischer Scientific) and western blotting was performed as previously described [50]. Primary antibodies were used; GAPDH (sc32233, 1:1000, Santa Cruz Biotechnology, Santa Cruz, CA), β-catenin (610154, 1:2000, Becton Dickinson, Frankilin Lakes, NJ), FOXA1 (ab170933, 1:1000, Abcam, Cambridge, MA, USA), Cytokeratin 20 (CK20) (ab76126, 1:10000, Abcam), Cytokeratin 5 (CK5) (PRB-160P, 1:1000, Covance).

### Quantitative real-time polymerase chain reaction (qPCR)

Total RNA was extracted from organoids using the RNeasy system (Qiagen, Hilden, Germany). Real-time qPCR was performed using the StepOnePlus system (Applied Biosystems, Foster City, CA, USA) as previously described [51]. TaqMan gene expression assays for AXIN2, KRT20 (CK20), KRT18 (CK18), KRT14 (CK14), KRT5 (CK5), and glyceraldehyde 3-phosphate dehydrogenase (GAPDH) were used. All primers were purchased from Thermo Fischer Scientific. GAPDH was used for normalization.

### Immunocytochemistry

For whole-mount immunostaining, organoids were fixed with 4% paraformaldehyde/PBS at room temperature for 15 minutes. Organoids were then blocked and permeabilized with Protein Block Serum-Free Ready-to-use (DAKO) containing 0.1% Triton X-100 at room temperature for 15 minutes. Organoids were incubated at 4° C with anti-β-catenin antibody (610154, 1:500, Becton Dickinson) overnight, then with Alexa-488 conjugated secondary antibody (Thermo Fischer Scientific) for 1 hour. After counterstaining with DAPI and F-actin probe (Thermo Fischer Scientific), organoids were mounted with ProLong Gold Antifade Reagent (Cell Signaling Technology, Danvers, MA). Images of fluorescent cells were obtained using a Zeiss Axio Observer microscope (Carl Zeiss AG, Oberkochen, Germany).

### Small interfering RNA transfection

For silencing CTNNB1, siRNAs for CTNNB1 or a scrambled siRNA control were purchased from Integrated DNA Technology (Coralville, IA, USA). Cell lines were transfected using Lipofectamine 2000 (Thermo Fisher Scientific) according to manufacture’s protocol. Efficiency of knockdown was tested by western blotting 48 hours after transfection. Transfected cells were subjected to proliferation assay, generation of organoids, and growth assay 48 hours after transfection.

### Statistical analysis

Statistical analysis was conducted using GraphPad Prism 7 (GraphPad Software, San Diego, CA, USA). Differences among multiple groups were compared by analysis of variance (ANOVA) followed by a Tukey’s multiple comparisons test. Two-group analysis was performed by unpaired *t*-test. A value of *p* < 0.05 was considered statistically significant.

## SUPPLEMENTARY MATERIALS FIGURES


