# Supplementary material for: Three-dimensional organoid culture reveals involvement of Wnt/β-catenin pathway in proliferation of bladder cancer cells
Source: Oncotarget. 2018 Jan 24;9(13):11060–70. doi: 10.18632/oncotarget.24308 (PMC5834271; doi:10.18632/oncotarget.24308)
Supplement: Supplementary file 1 [file oncotarget-09-11060-s001.pdf]

# Three-dimensional organoid culture reveals involvement of Wnt/β-catenin pathway in proliferation of bladder cancer cells

## SUPPLEMENTARY MATERIALS

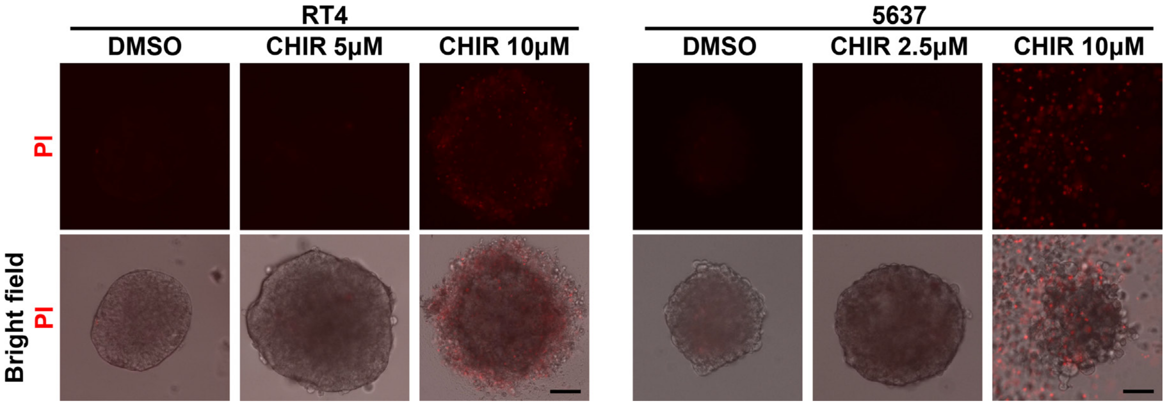


**Supplementary Figure 1: CHIR at high dose induces cell death in RT4- and 5637-derived organoids.** RT4- and 5637-derived organoids were treated with DMSO or CHIR of the indicated concentration for 5 days, and were incubated with propidium iodide (PI). Fluorescent images of PI and images merged with bright-field are shown. Scale bar, 100 μm.

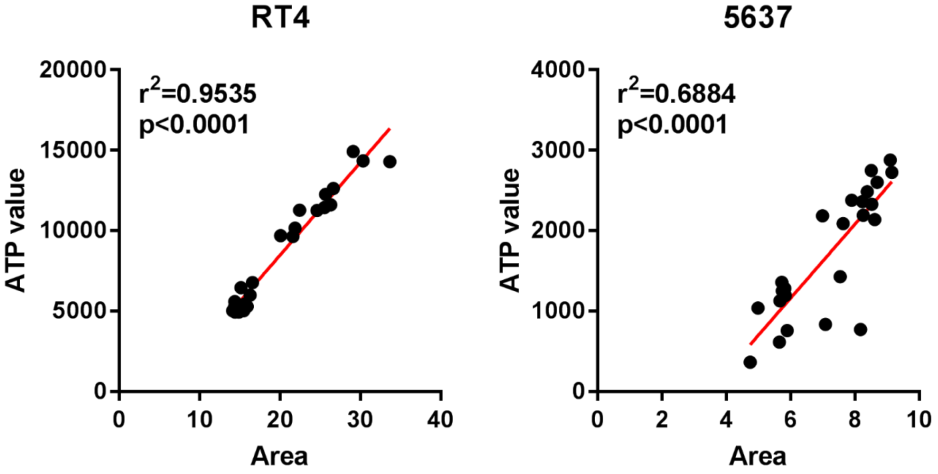


**Supplementary Figure 2: The ATP value of cell line-derived organoids correlates with the area after treatment of DMSO or CHIR.** RT4- and 5637-derived organoids were treated with DMSO or CHIR for 5 days, and ATP value of the organoids were measured. Correlation of the area and ATP value measured on the same day was examined by linear regression.

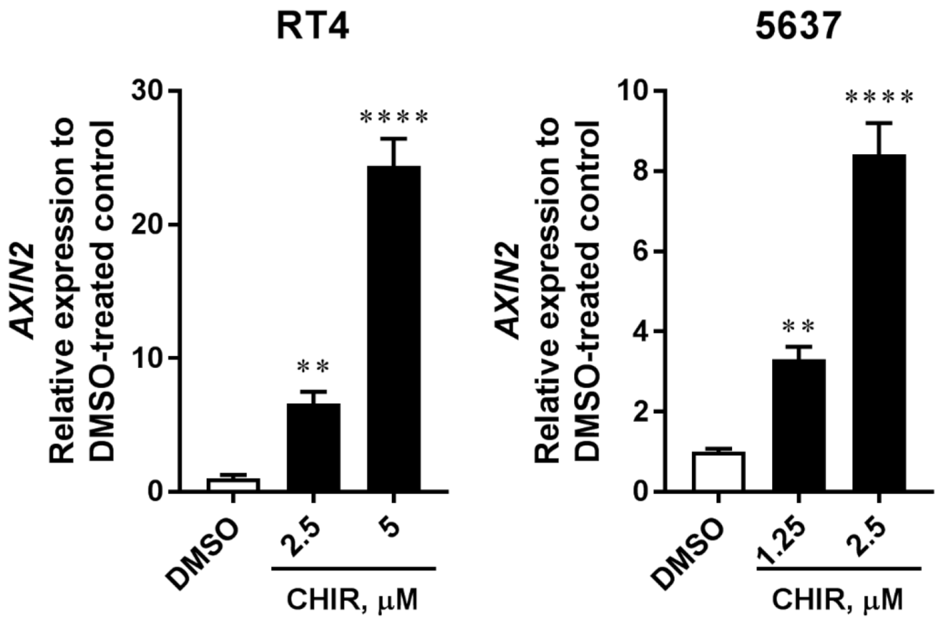


**Supplementary Figure 3: CHIR upregulates AXIN2 expression in RT4- and 5637-derived organoids in a dose-dependent manner.** Bar graphs show the relative gene expression of AXIN2 measured by qRT-PCR in RT4- and 5637-derived organoids treated with DMSO, or CHIR of the indicated concentration for 24 hours. GAPDH was used as a reference gene, and relative expression was calculated by the 2 ΔΔCT method. $^{**}P \leq 0.01$. $^{****}P \leq 0.0001$.

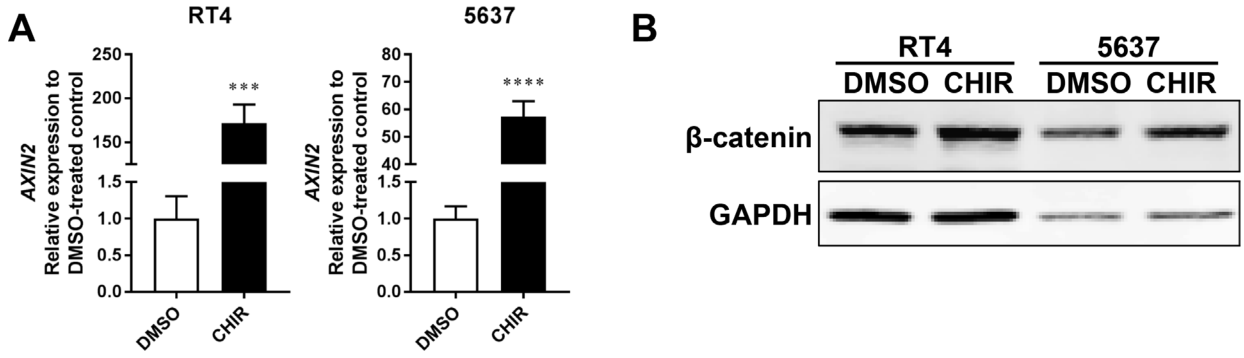


**Supplementary Figure 4: CHIR activates the Wnt/β-catenin pathway in bladder cancer cell lines in 2D adherent culture.** (**A**) Bar graphs show the relative gene expression of AXIN2 by qRT-PCR in RT4 and 5637 treated with DMSO, or 5 and 2.5 µM of CHIR for 24 hours in adherent culture, respectively. GAPDH was used as a reference gene, and relative expression was calculated by the 2 ΔΔCT method. (**B**) Western blot analysis shows protein levels of β-catenin and GAPDH in RT4 and 5637 treated with DMSO, or 5 and 2.5 µM of CHIR for 24 hours in adherent culture, respectively. GAPDH was used as a loading control. $^{***}P \leq 0.001$. $^{****}P \leq 0.0001$.

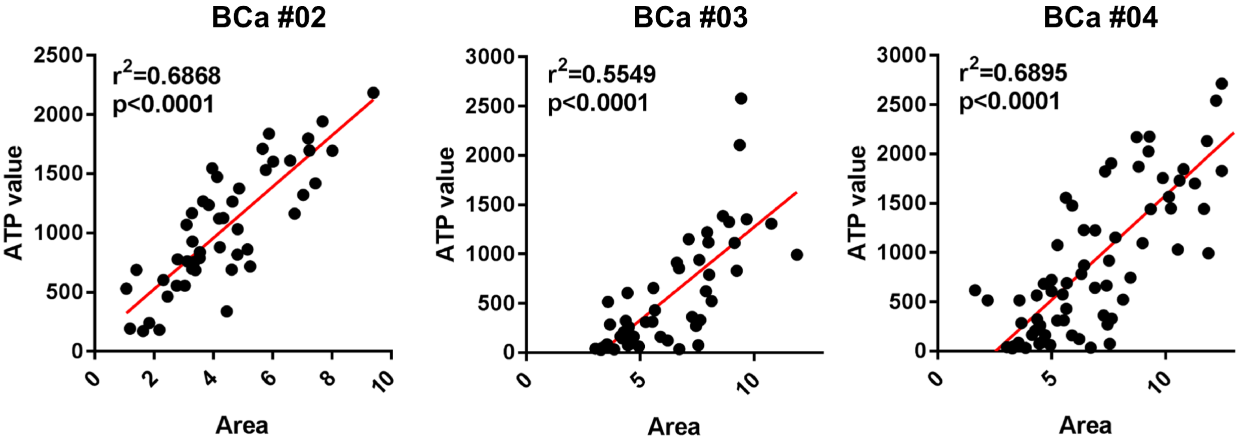


**Supplementary Figure 5: The ATP value of patient-derived organoids correlates with the area after treatment of DMSO or CHIR.** Organoids derived from 3 patient samples were treated with DMSO or CHIR for 5 days, and ATP value of the organoids were measured. Correlation of the area and ATP value measured on the same day was examined by linear regression.

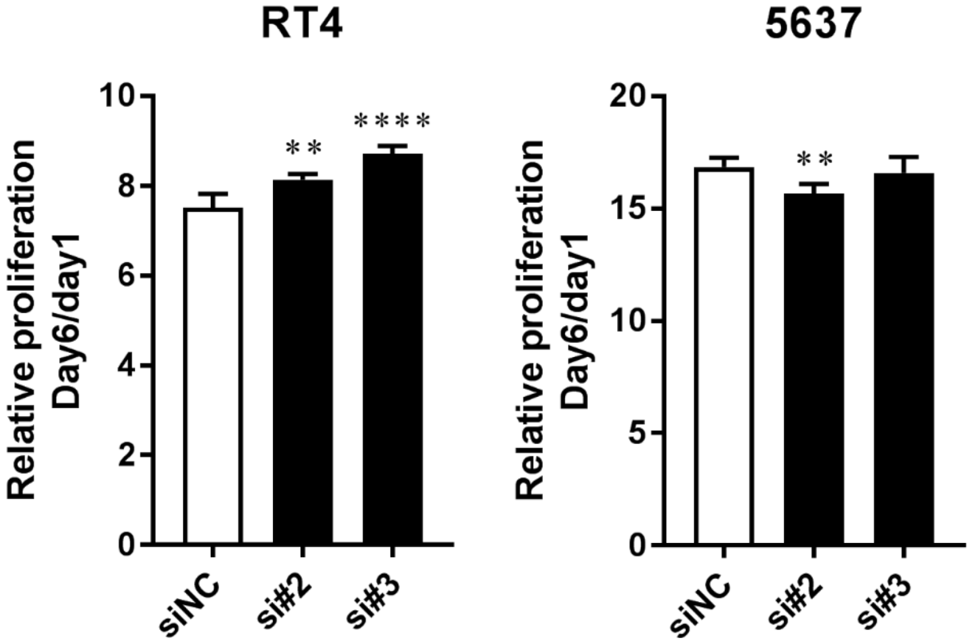


**Supplementary Figure 6: Gene silencing of β-catenin slightly affects proliferation of bladder cancer cells in 2D adherent culture.** RT4 and 5637 were transiently transfected with si-Control or si-CTNNB1. Transfected cells were plated in a 96 well plate. Viability of cells were measured using CellTiter-Glo Luminescent Cell Viability Assay (Promega) at 24 and 144 hours after seeding. Relative proliferation was calculated by dividing ATP value of 24 hours by that of 144 hours. $^{**}P \leq 0.01$. $^{****}P \leq 0.0001$.

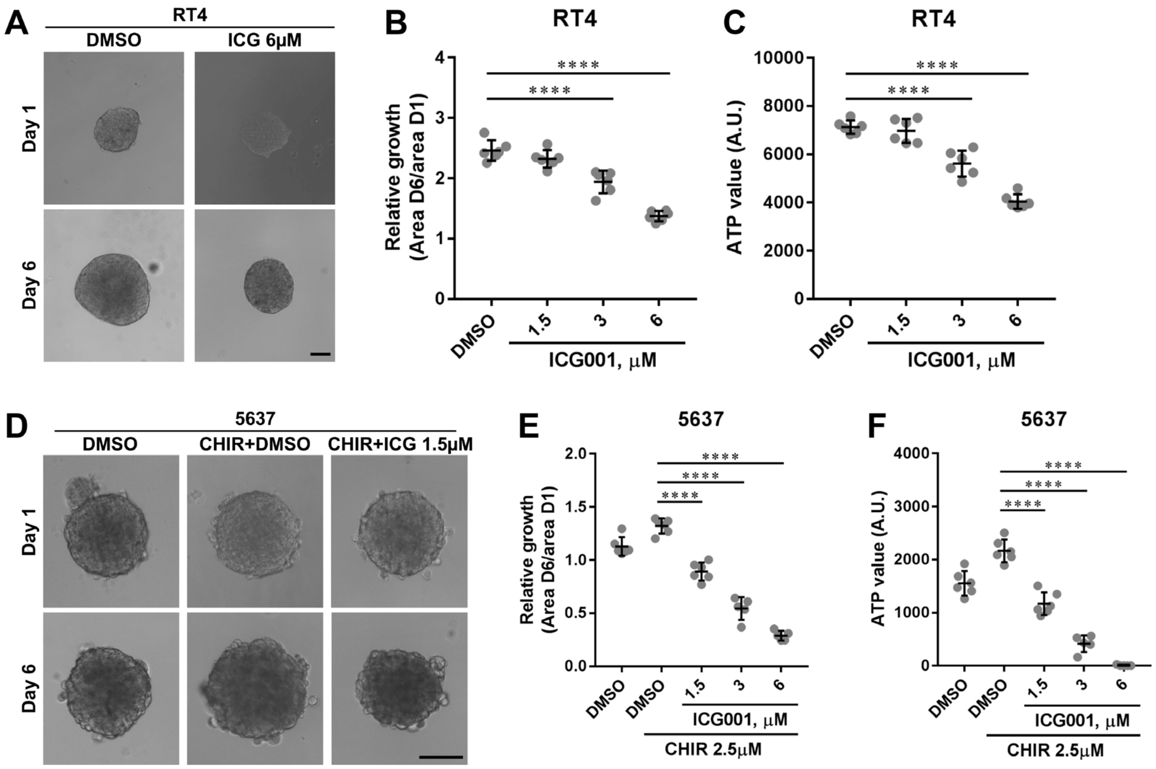


**Supplementary Figure 7: ICG-001 suppresses intrinsic growth of RT4-derived organoids and CHIR-induced growth of 5637- derived organoids.** (**A**) Representative images of RT4-derived organoids at day 1 and day 6 treated with DMSO or ICG-001 (Cayman Chemical) of the indicated concentration. Scale bar, 100 μm. (**B**) Relative growth of RT4-derived organoids was calculated by dividing area at day 6 by that at day 1, and depicted in the plot. (**C**) Viability of RT4-derived organoids at day 6 was measured by quantifying the amount of ATP, and depicted in the plot. (**D**) Representative images of 5637-derived organoids at day 1 and day 6 treated with DMSO, or CHIR and ICG001 of the indicated concentration. Scale bar, 100 μm. (**E**, **F**) Relative growth and viability of 5637-derived organoids at day 6 was depicted in the plot, respectively. $^{****}P \leq 0.0001$.

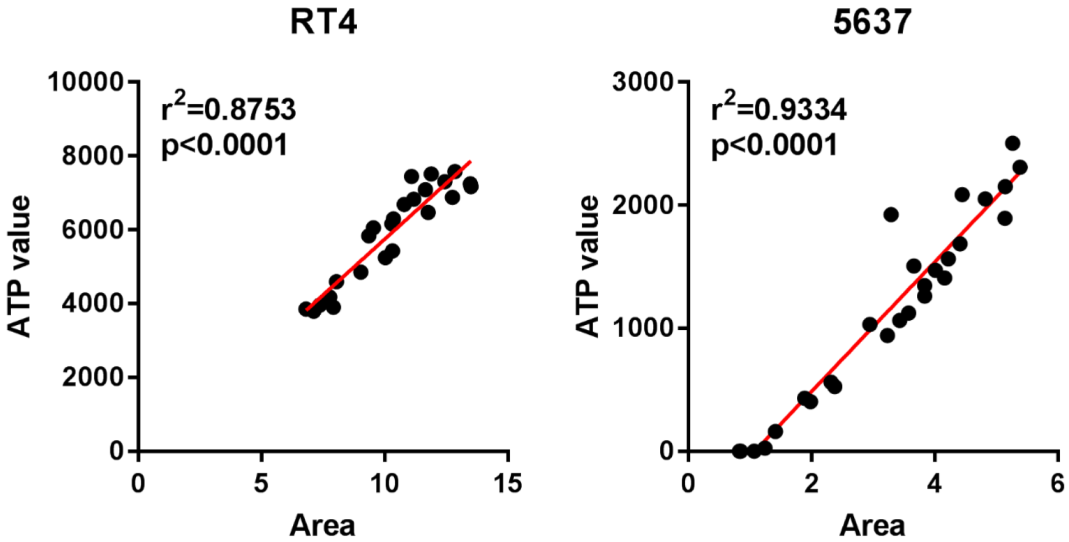


**Supplementary Figure 8: The ATP value of cell line-derived organoids correlates with the area after treatment of DMSO or ICG001.** RT4- and 5637-derived organoids were treated with DMSO or ICG001 for 5 days, and ATP value of the organoids were measured. Correlation of the area and ATP value measured on the same day was examined by linear regression.

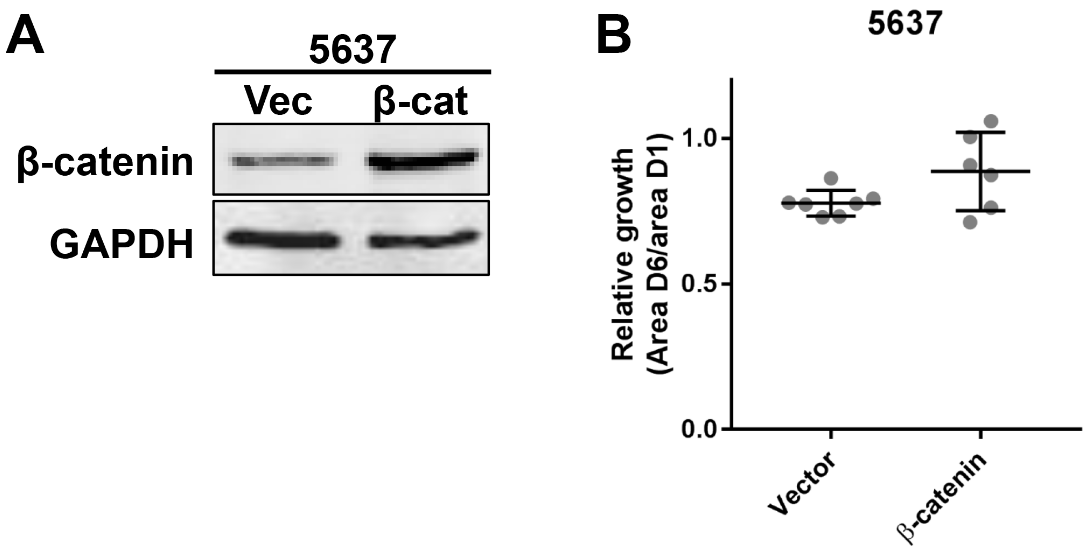


**Supplementary Figure 9: β-catenin overexpression does not enhance growth of bladder cancer organoids.** (**A**) 5637 was transfected with pCMV6-XL5 or pCMV6-CTNNB1 (SC107921, OriGene, Rockville, MD) using Lipofectamine 2000 (Thermo Fisher Scientific). Western blot analysis shows increased expression of β –catenin after transfection of the overexpressing plasmid. GAPDH was used as a loading control. (**B**) Images of organoids were captured on day 1 and 6. Relative growth was calculated by dividing area of day 6 by that of day 1. Each dot represents one organoid assessed.
